# Mitochondria and Other Organelles in Neural Development and Their Potential as Therapeutic Targets in Neurodegenerative Diseases

**DOI:** 10.3389/fnins.2022.853911

**Published:** 2022-04-05

**Authors:** Shuyuan Zhang, Juan Zhao, Zhenzhen Quan, Hui Li, Hong Qing

**Affiliations:** ^1^Key Laboratory of Molecular Medicine and Biotherapy, School of Life Science, Beijing Institute of Technology, Beijing, China; ^2^Aerospace Medical Center, Aerospace Center Hospital, Beijing, China

**Keywords:** neural development, mitochondira, metabolic reprogramming, neurodegenerative disease, antioxidants

## Abstract

The contribution of organelles to neural development has received increasing attention. Studies have shown that organelles such as mitochondria, endoplasmic reticulum (ER), lysosomes, and endosomes play important roles in neurogenesis. Specifically, metabolic switching, reactive oxygen species production, mitochondrial dynamics, mitophagy, mitochondria-mediated apoptosis, and the interaction between mitochondria and the ER all have roles in neurogenesis. Lysosomes and endosomes can regulate neurite growth and extension. Moreover, metabolic reprogramming represents a novel strategy for generating functional neurons. Accordingly, the exploration and application of mechanisms underlying metabolic reprogramming will be beneficial for neural conversion and regenerative medicine. There is adequate evidence implicating the dysfunction of cellular organelles—especially mitochondria—in neurodegenerative disorders, and that improvement of mitochondrial function may reverse the progression of these diseases through the reinforcement of adult neurogenesis. Therefore, these organelles have potential as therapeutic targets for the treatment of neurodegenerative diseases. In this review, we discuss the function of these organelles, especially mitochondria, in neural development, focusing on their potential as therapeutic targets in neurodegenerative disorders, including Alzheimer’s disease, Parkinson’s disease, Huntington’s disease, and amyotrophic lateral sclerosis.

## Introduction

Neural development is a complex process that requires various endogenous and exogenous signals to generate connected neural circuits with specific functions. During embryonic neurogenesis, the folding of the neural plate forms the neural tube, followed by the formation of temporarily and spatially regulated morphogen gradients along the anterior–posterior (A–P) and dorsal–ventral (D–V) axes (Tao and Zhang, 2016). In A–P patterning, WNT, fibroblast growth factor (FGF), and retinoic acid (RA) gradients predominate in forebrain, mid-hindbrain, and spinal cord segmentation; while WNT, bone morphogenetic protein (BMP), and sonic hedgehog (Shh) gradients regulate D–V patterning, i.e., for ventralization and dorsalization of the developing nervous system (Tao and Zhang, 2016). Once brain tissue maturation is complete, the number of neural stem cells (NSCs) decreases, maintaining multipotency, i.e., the ability to differentiate into neurons, astrocytes, and oligodendrocytes in limited regions of the brain.

**FIGURE 1:**
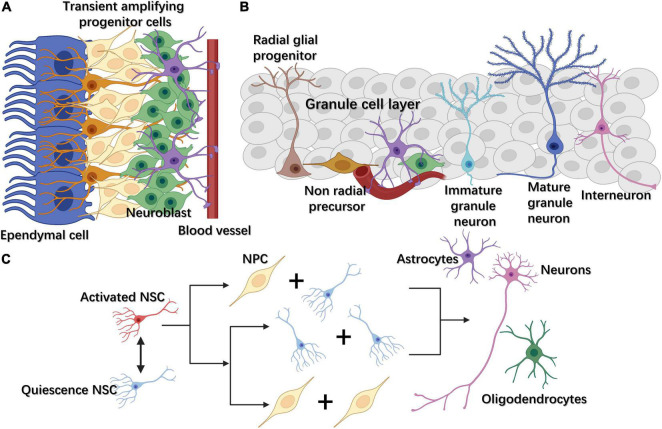
**(A)** In the subventricular zone (SVZ), NSCs, also called radial glia-like cells, gradually differentiate into transient amplifying progenitor cells and neuroblasts. **(B)** In the subgranular zone (SGZ), radial glia-like cells differentiate into non-radial progenitor cells, intermediate progenitor cells (IPCs), and neuroblasts. The latter become mature excitatory granule neurons, which gradually migrate to the granule cell layer and extend their dendrites toward the molecular layer in the hippocampus. **(C)** Adult neural stem cells (NSCs) can exit or enter the cell cycle through changing between quiescence and activated states. Once activated, NSCs can divide either symmetrically or asymmetrically. Neural progenitor cells (NPCs) can further differentiate into three cell types: neurons, astrocytes, and oligodendrocytes.

NSCs are located in two main neurogenic niches in the adult mammalian brain, namely, the subventricular zone (SVZ) lining the lateral ventricles and the subgranular zone (SGZ) within the dentate gyrus (DG) of the hippocampus (Figures 1A,B; Bond et al., 2015). NSCs remain quiescent until they receive activation signals, when they then enter the cell cycle. After activation, NSCs can perform two types of division, symmetric or asymmetric. In the former, NSCs divide into two NSCs or two progenitor cells, while in asymmetric division they divide into an NSC and a progenitor cell (Figure 1C; Bond et al., 2015). The progenitor cells will later proliferate, migrate, differentiate into specific cell types, and integrate into the neural circuits (Vieira et al., 2018). These processes are regulated by a variety of endogenous and exogenous cues, including growth factors, morphogen-mediated signaling pathways, transcription factors, extracellular matrix (ECM), glial cells, and perineuronal nets (PNNs) (Vieira et al., 2018; Cope and Gould, 2019).

To date, various methods have been developed to investigate neurogenesis *in vitro*. Forced expression of neural lineage-determining transcription factors; treatment with small molecules, growth factors, and neurotrophic factors; and even three-dimensional (3D) cell culture methods have all been applied to induce functional neurons from pluripotent stem cells (PSCs) or somatic cells (Yang et al., 2011; Gascon et al., 2017; Gong et al., 2018; Vieira et al., 2018). An alternative method with huge potential for cell lineage regulation that has also been explored is metabolic reprogramming. Cellular metabolism can influence cell proliferation and lineage commitment by promoting the epigenetic modification of histones and DNA (Ly et al., 2020). Additionally, during somatic cell reprogramming, cells undergo a metabolic shift from oxidative phosphorylation (OXPHOS) to glycolysis, accompanied by changes in the morphology and number of mitochondria (Nishimura et al., 2019). Importantly, the forced expression of LIN28A can activate pyruvate dehydrogenase kinase 1 (PDK1)-mediated glycolysis/tricarboxylic acid (TCA)/OXPHOS uncoupling, thus inducing metabolic reprogramming from OXPHOS to glycolysis and the reprogramming of mitochondria into a fused, more functional state in somatic stem cells (SSCs) (Pieknell et al., 2021). This process may enhance the self-renewal and differentiation potential of SSCs in regenerative medicine (Pieknell et al., 2021). Therefore, focusing on the potential of various organelles and their mechanistic roles in neurogenesis could expand the repertoire of methods for neurogenesis *in vitro*, especially through metabolic reprogramming.

There is adequate evidence supporting that organelle dysfunction is strongly associated with neurodegenerative disorders, such as Alzheimer’s disease (AD) and Parkinson’s disease (PD). Mitochondria dysfunction is found in AD patients, including disrupted mitochondrial bioenergetics, disturbed mitochondrial genomic homeostasis, and increased oxidative stress (Wang et al., 2020). Mitochondria-related dysfunction is also involved in PD, mainly through respiratory chain impairment, mitochondrial DNA (mtDNA) alterations, and dysregulation of mitophagy, through mutations in Parkin and PTEN-induced kinase 1 (PINK1) in familial PD (Monzio Compagnoni et al., 2020). Moreover, studies have suggested that mitochondrial dysfunction may play an important role in AD and PD *via* the dysregulation of adult neurogenesis (Richetin et al., 2017; Singh et al., 2018). Other organelles such as the ER, lysosomes, and endosomes are also correlated with neurodegenerative diseases (Hetz and Saxena, 2017; Pensalfini et al., 2020; Root et al., 2021).

In this review, we focus on the function of organelles, including mitochondria, endoplasmic reticulum (ER), lysosomes, and endosomes, in neural development, and discuss their potential as therapeutic targets in neurodegenerative diseases.

## Mitochondria

Mitochondria play a central role in energy production and metabolic regulation, as well as apoptosis and autophagy (Nunnari and Suomalainen, 2012). Evidence has accumulated implicating mitochondria in neural development, with studies reporting that mitochondria can regulate neurogenesis and neuroplasticity through metabolic conversion, calcium regulation, apoptosis, autography, and mitochondrial dynamics. Thus, mitochondria participate in various aspects of neural differentiation, such as neurite growth, dendrite remodeling, and neurotransmitter release (Cheng et al., 2010). Here, we review the role of mitochondria in neurogenesis, focusing on metabolic transformation, reactive oxygen species (ROS) production, mitochondrial dynamics, mitophagy, apoptosis, calcium release, and the interaction with other organelles, especially the ER.

### Metabolic Conversion and Reactive Oxygen Species Production

Mitochondria change their metabolic pattern from glycolysis to OXPHOS during neurogenesis. In murine embryonic stem cells (ESCs), high glycolytic flux, low mitochondrial oxygen consumption, and elevated activity of glycolytic enzymes can lead to a high proliferative capacity (Kondoh et al., 2007). When human somatic cells are reprogrammed into human induced pluripotent stem cells (iPSCs), the mitochondria-related oxidative stress pathway is suppressed, but is then reactivated after spontaneous differentiation (Prigione et al., 2010). The repression of the mitochondrial respiratory chain through the inhibition of mitochondrial complex III can enhance the pluripotency of human ESCs, which blocks neural differentiation (Varum et al., 2009; Pereira et al., 2013). Moreover, human iPSCs display a similar metabolic pattern to that of human ESCs, i.e., they mainly rely on glycolysis for their energy requirements (Prigione et al., 2010). Moreover, it has been shown that spontaneous differentiation is reduced when human ESCs are cultured in hypoxic conditions, which also increases embryonic body (EB) formation (Ezashi et al., 2005). During ESC differentiation, there is an increase in ATP and ROS production, as well as antioxidant enzyme expression (Cho et al., 2006). There is also a switch from glycolysis to OXPHOS when human neural progenitor cells (NPCs) differentiate into motor neurons, along with increased rates of ATP synthesis and mitochondrial biogenesis (O’Brien et al., 2015). The results of these studies showed that a correlation exists between mitochondria and neurogenesis, in that mitochondria-meditated metabolic shifts can contribute to neural differentiation.

The mechanisms underlying how mitochondria regulate neurogenesis through metabolic transformation remain poorly understood. Uncoupling protein (UCP), localized to the mitochondrial inner membrane where it induces proton leakage from the mitochondrial intermembrane space to the matrix, can uncouple OXPHOS and downregulate ATP production (Sreedhar and Zhao, 2017). UCP2, a member of the UCP family, was reported to be inhibited during the differentiation of human PSCs, leading to reduced glycolysis and a metabolic shift to OXPHOS (Zhang et al., 2011). This result indicates that UCP might play a role in metabolic reprogramming, inducing cell reprogramming through promoting a metabolic switch to glycolysis. Blocking mitochondrial metabolism can also lead to impaired neural development. In one study, cultured adult neural stem/progenitor cells (NSPCs) were treated with rotenone and oligomycin to interrupt ETC and OXPHOS activity, respectively, and both treatments disrupted cell proliferation and significantly increased cell death (Beckervordersandforth et al., 2017). The authors then conditionally ablated mitochondrial transcription factor A (TFAM) to disrupt ETC and OXPHOS activity using *Tfam*^cko^ mice, and found that this exerted only a limited effect on the number of activated NSCs; however, the proliferation of intermediate progenitor cells (IPCs) was markedly decreased in the animals, suggesting that ETC and OXPHOS activity might play a major role in IPC proliferating, including in further lineage progression, neural differentiation, and maturation (Beckervordersandforth, 2017; Beckervordersandforth et al., 2017). For instance, treatment of branched-chain amino acids, TCA intermediates, and co-factors to IPCs increased oxidative metabolism, which subsequently enhanced ATP production and activated the mTORC1 complex (Bifari et al., 2020). Moreover, the sustained activation of mTORC1 led to a decline in NSC proliferation while enhancing NSC differentiation, as evidenced by increased dendritic branching, greater numbers of presynaptic vesicles, and the presence of mature spine types (Bifari et al., 2020). A different study found that signal transducer and activator of transcription 3 (STAT3) promoted the expression of mitochondrial OXPHOS-related genes, while the conditional knockdown of STAT3 could downregulate the mTOR pathway and promote the proliferation of neural progenitors, suggesting that the STAT3-mitochondria metabolism axis could play a role in facilitating neural differentiation (Su et al., 2020). Mitochondrial oxidative metabolism also functions in the regulation of neuron morphology and maturation. The knockout of cytoplasmic polyadenylation element-binding protein 1 (CPEB1) in mice resulted in impaired translation of NDUFV2, an ETC complex I protein, which led to reduced ATP production and defective dendrite morphogenesis in hippocampal neurons (Oruganty-Das et al., 2012).

**FIGURE 2:**
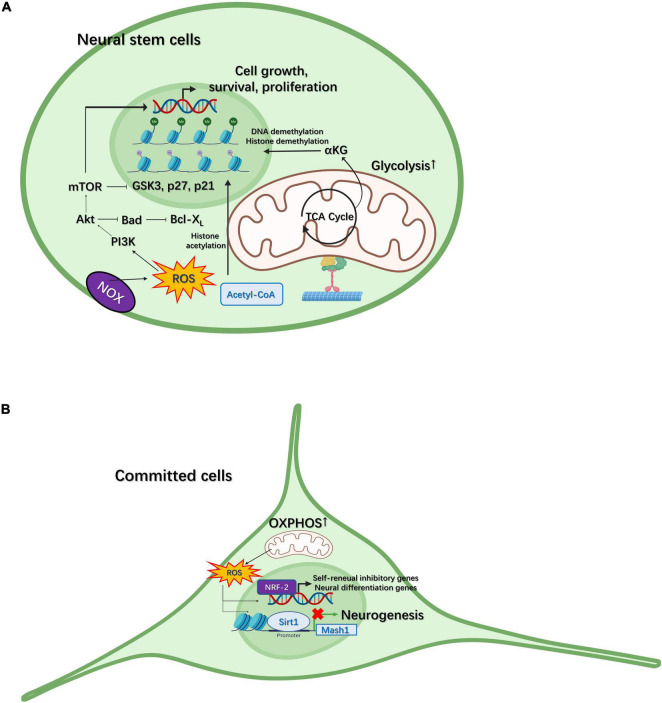
Mitochondria participate in neural differentiation in neural stem cells (NSCs) and committed cells. **(A)** Intermediate metabolites generated in the tricarboxylic acid (TCA) cycle, such as α–ketoglutarate (αKG), can regulate neurogenesis through histone/DNA demethylation for the maintenance of stem cell pluripotency. Reactive oxygen species (ROS) generated through NADPH-oxidase (NOX) can regulate the proliferation of NSCs *via* the PI3K/AKT pathway. **(B)** In committed cells, the metabolic pattern gradually switches from glycolysis to oxidative phosphorylation (OXPHOS). ROS generated through the electron transport chain (ETC) can activate self-renewal-inhibiting and neural differentiation-promoting genes *via* NRF2-dependent retrograde signaling. In contrast, ROS can also facilitate the SIRT1-mediated inhibition of *Mash1* expression, thereby guiding NPCs to an astroglial fate.

During this metabolic change from glycolysis to OXPHOS, increased ROS production can also facilitate neural differentiation to some extent. For example, ROS can enhance neurotrophic factor signaling, such as that mediated by brain-derived neurotrophic factor (BDNF), to regulate the self-renewal of NSCs and neurogenesis *via* phosphoinositide 3-kinase (PI3K)/AKT signaling (Figure 2A; Le Belle et al., 2011). ROS signaling can also activate differentiation-related genes in NSCs through nuclear factor erythroid-2-related factor 2 (NRF2)-dependent retrograde signaling (Khacho et al., 2016), implying that ROS can activate nuclear transcriptional programming (Figure 2B). Additionally, ROS promotes the differentiation of NPCs toward the astrocytic lineage. Under oxidative conditions, Sirt1 and Hes1 form a complex at the *Mash1* promoter, leading to the deacetylation of H3K9 (Figure 2B; Prozorovski et al., 2008). This subsequently guides NPCs toward an astroglial lineage instead of a neural lineage. ROS can also regulate the cell cycle in NSCs. ROS-meditated oxidation of proteins—such as protein kinases [e.g., receptor tyrosine kinases (RTKs), mitogen-activated protein kinase (MAPK), and mechanistic target of rapamycin (mTOR)] and transcription factors [e.g., p53, NRFs, and forkhead transcription factors of the O-class (FOXOs)]—plays an essential role in stem cell fate decision (Tan and Suda, 2018). When complex I of the ETC is disrupted, mitochondria can also influence the G1/S cell cycle checkpoint in a ROS or ATP concentration-dependent manner. In the eye imaginal disc of Drosophila, high ROS levels lead to the upregulation of cyclin E-CDK2 inhibition through apoptosis signal-regulating kinase 1 (Ask1), (c-Jun N-terminal kinase) JNK, and FOXO (Owusu-Ansah et al., 2008), while low ATP concentrations can downregulate cyclin E levels through the activation of AMP-activated protein kinase (AMPK) and p53 (Owusu-Ansah et al., 2008). ROS concentrations can also modulate the quiescence and activation of adult mouse hippocampal NSCs. For example, Nes-GFP^+^ cells with high ROS levels remain quiescence and show sparse EdU incorporation, whereas, with reduced levels of ROS, the expression of markers associated with cell cycle activation and neural commitment is increased in these cells (Adusumilli et al., 2021).

Intermediate metabolites generated in the TCA cycle can also modulate stem cell fate (Figure 2A). For example, in naïve mouse ESCs, a high α–ketoglutarate (αKG)/succinate ratio can induce histone/DNA demethylation, which subsequently maintains the pluripotency of stem cells and inhibits their differentiation (Carey et al., 2015). There is also evidence that acetyl-CoA can promote histone acetylation and maintain the pluripotency of mouse and human ESCs (Moussaieff et al., 2015). Moreover, the addition of acetate, a precursor of acetyl-CoA, delays cell differentiation in a dose-dependent manner (Moussaieff et al., 2015). These observations indicate that mitochondria-mediated metabolism plays a significant role in stem cells not only by providing carbon precursors to increase biomass for the generation of phospholipids, amino acids, and nucleotides to guarantee stem cell growth, but also by regulating the epigenetic signature for stem cell fate decisions (Ly et al., 2020).

### Mitochondrial Dynamics

During neurogenesis, mitochondrial dynamics, regulated by a balance between mitochondria fusion and fission, contribute to neural differentiation in various ways. Mitochondria fusion, mainly mediated by mitofusins 1 and 2 (MFN1/2) and optic atrophy protein (OPA1), allows mitochondria to exchange membranous materials as well as the rescue of damaged mitochondria, while the fission process, regulated by dynamin-related protein 1 (DRP1) and fission 1 (FIS1), serves to remove and degrade mitochondria with damaged DNA (Son and Han, 2018). During neural differentiation, the morphology of mitochondria changes. Mitochondria exhibit an elongated morphology in Sox2-expressing, uncommitted NSCs from mouse E15.5 cortex, become fragmented in Tbr2-expressing committed NPCs, and then return to an elongated state in differentiated, Tuj1-expressing post-mitotic neurons (Khacho et al., 2016). Importantly, after NSC mitosis, the mitochondria that undergo fusion in the daughter cells are fated for self-renewal, but tend toward fission when the daughter cells are destined to become neurons (Iwata et al., 2020). Additionally, the downregulation of OPA1 leads to fragmented mitochondria, reduced expression of components of mitochondrial respiratory complexes, decreased mitochondrial membrane potential, restricted dendritic growth, and downregulation of synaptic protein expression in rat cortical primary neurons (Bertholet et al., 2013). Thus, mitochondria contribute to whether NSCs undergo self-renewal or neural differentiation after cell division. Furthermore, a fragmented mitochondrial phenotype in NPCs results in increased ROS signaling, thereby modulating nuclear transcriptional programs through retrograde signaling (Khacho et al., 2016).

### Mitophagy

Mitochondria dynamics also have a role in the repair of damaged mitochondria when cells undergo oxidative stress resulting from increased ROS production. Impaired mitochondria are degraded through mitophagy (Yoo and Jung, 2018), and this mitophagy process can also influence neural fate determination. Studies have shown that programmed mitophagy, regulated by hypoxia and the hypoxia-inducible factor-1 (HIF-1) target gene BCL2/adenovirus E1B 19 kDa protein-interacting protein 3-like (*BNIP3L*)/*NIX*, can promote metabolic reprogramming toward glycolysis, thereby inducing retinal ganglion cell (RGC) differentiation (Esteban-Martinez et al., 2017). Although this contrasts with the previous finding that differentiation of most cell types is typically related to enhanced mitochondrial activity, this contradiction can be explained by the fact that RGCs have enhanced metabolite requirements when they begin to elongate their axons to connect to the brain, resulting in a temporary reduction in mitochondrial numbers and an increase in glycolysis (Esteban-Martinez et al., 2017). In addition, BNIP3L-mediated mitophagy can promote the differentiation of oligodendrocyte progenitor cells (OPCs) into oligodendrocytes. In support of this, the knockdown of the pro-autophagic proteins ATG9A and BECN1 was shown to reduce OPC survival and proliferation, while autophagy flux was increased during differentiation (Yazdankhah et al., 2021). Furthermore, mitophagy and mitochondria remodeling is involved in NGF-induced neural differentiation (Martorana et al., 2018).

### Apoptosis

Under conditions of severe stress, the mitochondrial apoptosis pathway is activated instead of mitophagy. During this process, multiple stimuli, including oxidative stress, Ca^2+^ overload, and cytotoxic agents, can trigger the oligomerization of Bax and Bak, pro-apoptotic proteins belonging to the Bcl-2 family, leading to the permeabilization of the mitochondrial outer membrane and release of cytochrome *c* (Czabotar et al., 2014). Cytochrome *c*, together with Apaf-1 and pro-caspase-9, forms a multiprotein apoptosome, leading to the activation of caspase-9 and the subsequent initiation of the apoptotic cascade (Green and Reed, 1998; Sinha et al., 2013). Bcl-xL, a pro-survival protein within the Bcl-2 family, can bind to Bax and retrotranslocate it to the cytoplasm and stabilize its inactive form, thereby inhibiting its autoactivation at the mitochondrial outer membrane (MOM) (Edlich et al., 2011; Martinou and Youle, 2011). Bcl-xL can also promote the proliferation of neural progenitors, thus simultaneously inducing neuron generation and blocking that of glia in human NSCs (Liste et al., 2007). There is also evidence that Bcl-xL can enhance the spontaneous differentiation of human NSCs into dopaminergic neurons, but not into other neural cell types (Liste et al., 2004). Furthermore, in Bax knockout mice, astrocyte generation was found to be decreased in developing cortices, and overexpressing Bax and Bcl-xL promotes the conversion of embryonic cortical precursors to astrocytes and neurons, respectively (Chang et al., 2007). Furthermore, it has been shown that when Bcl-xL is overexpressed in cultured E14 cortical precursors, the expression of pro-neuronal basic helix-loop-helix (bHLH) transcription factors, including Mash1, neurogenin-1 (Ngn1), and NeuroD1, is upregulated, while that of anti-neuronal bHLH transcription factors such as Hes-1 and -5 and Id-1, -2, and -3, is downregulated, with the opposite results being observed in Bax-overexpressing cells (Chang et al., 2007). The overexpression of Bax or Bcl-xL can also change the phosphorylation state of MAPK, AKT, STAT1 and 3, and cAMP-response element-binding protein (CREB) (Chang et al., 2007). Therefore, the apoptosis-related proteins in mitochondria, especially those of the Bcl-2 family, are involved in neural proliferation, differentiation, and neural-vs.-astroglial fate determination. Although ROS generation, LC3-II lipidation, mitochondrial membrane permeabilization, and cytochrome *c* release all occur during the early stage of NSC differentiation in the mouse, p53 can translocate to mitochondria to reduce oxidative stress, mitophagy, and apoptosis, which can also enhance neural lineage induction (Xavier et al., 2014).

### Mitochondria–Endoplasmic Reticulum Interaction

The interaction between mitochondria and other organelles merits substantial research attention. This is especially true for the ER, which can modulate Ca^2+^ signaling during neurogenesis. For instance, Bcl-xL can prolong the cell cycle, thereby increasing the numbers of intermediate progenitors (IPs), which amplifies the pool of neuronal progeny (Garcia-Garcia et al., 2012). In human NSCs, Bcl-xL can directly bind and modulate the sensitivity of the inositol trisphosphate (inositol 1,4,5-trisphosphate; IP_3_) receptor at the ER membrane, and then enhance spontaneous Ca^2+^ release from the ER, which subsequently upregulates p53, a transcriptional regulator of p21, a negative modulator of cell cycle progression (Garcia-Garcia et al., 2012). When phosphorylated by Polo kinase, mitochondrial rho GTPase (Miro), located on the MOM, can regulate the integrity of the ER–mitochondria contact site (ERMCS) and interact with Ca^2+^ transporters at the ERMCS, thus regulating mitochondrial Ca^2+^ homeostasis through the transportation of Ca^2+^ from the ER to mitochondria (Lee et al., 2016). This process was found to benefit NSC maintenance and lineage progression (Lee et al., 2016). Moreover, in PINK mutant dopaminergic neurons in a Drosophila model of PD, the ERMCS is strengthened, and the transportation of Ca^2+^ from the ER to mitochondria is increased (Lee et al., 2018), leading to elevated mitochondrial Ca^2+^ and impaired neural function and survival (Lee et al., 2018).

### Mitochondria and Neuroplasticity

Mitochondria also play an important role in regulating neuroplasticity. Mitochondria are distributed along axons, dendritic shafts, and presynaptic terminals (Cheng et al., 2010). Mitochondria have been observed to stop and localize at synapses and branch points of dendrites after neural maturation (Faits et al., 2016). This movement is mediated through the linkage of Miro-1 to KIF5 motor proteins, which can transport mitochondria *via* microtubules to where ATP is required in neurons (Macaskill et al., 2009). High Ca^2+^ levels inhibit the interaction between Miro-1 and KLF5, allowing the activation of glutamate receptors and the recruitment of mitochondria to activated synapses (Macaskill et al., 2009). At presynapses, mitochondria can regulate neurotransmission mainly through ATP provision and the inhibition of Ca^2+^-mediated signaling (Todorova and Blokland, 2017). ATP generated by mitochondria is used for vesicular exocytosis, endocytosis, and recycling as well as the reversal of ion fluxes through ion channels to facilitate synaptic transmission (Devine and Kittler, 2018). Through the use of an optical reporter of presynaptic ATP, *Syn-ATP*, it was shown that electrical activity consumed a large quantity of ATP, synthesized both *via* glycolysis and OXPHOS, with vesicle cycling accounting for the largest proportion of the ATP consumed (Rangaraju et al., 2014). Additionally, Ca^2+^ released from mitochondria can interact with calmodulin and modulate RAB GTPases activity, thereby inhibiting vesicle exocytosis and promoting the recruitment of synaptic vesicles from the readily releasable pool (RRP) (Sakaba and Neher, 2001; Devine and Kittler, 2018). While in the postsynaptic dendrites, mitochondria exist in temporally stable compartments provide energy for local protein synthesis and regulate synaptic plasticity (Rangaraju et al., 2019). There is also evidence that mitochondria participate in neurite outgrowth. The involvement of mitochondria-located STAT3 mono-phosphorylated at residue serine (727) (P-Ser-STAT3) in NGF-induced neurite outgrowth was discovered in PC12 cells (Zhou and Too, 2011). Importantly, mitochondria play a role in the regulation of axon branching through branching-promoting signals, such as nerve growth factors (NGFs), or branching-inhibiting signals, including chondroitin sulfate proteoglycans (CSPGs), components of the extracellular matrix (Smith and Gallo, 2018). These promotive or inhibitory signals can result in hyperpolarization or depolarization, respectively, to strengthen or attenuate mitochondrial respiration and facilitate or repress mitochondria-associated actin dynamics and intra-axonal translation of branching-promoting actin regulatory proteins (Verburg and Hollenbeck, 2008; Sainath et al., 2017; Smith and Gallo, 2018). Regarding dendrite regulation, PINK1 in the cytosolic pool participates in the regulation of dendrite morphology through enhanced anterograde transportation of dendritic mitochondria (Dagda et al., 2014). Mechanistically, the activity of protein kinase A (PKA) downstream of PINK1 was enhanced, as well as the binding of the regulatory subunit β of PKA to dual-specificity A kinase anchoring protein 1 (D-AKAP1), known as the PKA-mitochondrial scaffold (Das Banerjee et al., 2017). Furthermore, the D-AKAP1/PKA complex promoted the phosphorylation of Miro-2 to control mitochondria trafficking in dendrites (Das Banerjee et al., 2017). It was also found that the interruption of mitochondrial transportation to dendrites can disturb dendritic outgrowth in mouse Purkinje cells (Fukumitsu et al., 2015). To be specific, ATP generated by dendritic mitochondria and the creatine kinase/phosphocreatine (CK/PCr) shuttle system supports cofilin-mediated actin turnover and dendrite extension in growing mouse Purkinje cells (Fukumitsu et al., 2015). Besides ATP, mitochondrial calcium homeostasis can also involve in control of dendritic growth. As reported, cyclophilin D (CypD) mediates the opening of mitochondrial permeability transition pore (mPTP), which subsequently regulates intra-dendritic calcium dynamics through promotion of calcium release and facilitates activity-induced dendritic protrusion outgrowth (Sui et al., 2018).

## Other Organelles

### Endoplasmic Reticulum

As mentioned before, the ER can regulate neurogenesis through the modulation of mitochondrial Ca^2+^ homeostasis *via* the ERMCS (Lee et al., 2016). Additionally, given its function as a dynamic calcium store, the ER controls calcium metabolism by responding to cellular signals for Ca^2+^ release. When the intracellular Ca^2+^ level is low, IP_3_ binds to IP_3_R to mediate Ca^2+^ release from the ER to the cytoplasm, whereas when the Ca^2+^ concentration in the ER is low, store-operated Ca^2+^ entry (SOCE) is activated. During this process, clustered stromal interacting molecule-1 (STIM1) proteins on the ER membrane trap Orai subunits on the plasma membrane, leading to the formation of Ca^2+^ release-activated channels (CRAC) for the entry of extracellular Ca^2+^ into the ER lumen to restore Ca^2+^ levels (Schwarz and Blower, 2016). This replenishment of intracellular Ca^2+^ levels can promote cell cycle elongation and an increase in IP numbers, thereby also boosting the numbers of neurons (Garcia-Garcia et al., 2012). In addition, the knockdown or repression of STIM1 and Orai, molecular components of the SOCE machinery, result in the inhibition of NPC proliferation (Li et al., 2012; Somasundaram et al., 2014; Gopurappilly et al., 2018), while STIM1 may also be involved in early neural differentiation (Hao et al., 2014; Gopurappilly et al., 2018). These effects may be associated with a role of SOCE in cell cycle regulation as knockdown of transient receptor potential canonical 1 (TRPC1), a component of storage-operated Ca^2+^ channels (SOE), leads to mouse hippocampal NPC cell cycle arrest at the G1/S transition (Li et al., 2012). Furthermore, gene array assays showed that the levels of CDK inhibitor 2A were upregulated and those of Bcl-2 decreased after TRPC1 knockdown (Li et al., 2012).

Several ER-resident proteins are associated with neurite outgrowth and neural migration. For example, the deletion of mesencephalic astrocyte-derived neurotrophic factor (MANF), an ER-resident protein, results in impaired neural differentiation and neurite extension in mouse NSCs as well as delayed neuronal migration in the developing cortex, mainly by increasing ER stress and promoting unfolded protein response (UPR)-mediated signaling (Tseng et al., 2017). During neurite extension, there is a greater requirement for protein synthesis, which triggers the UPR to restore protein homeostasis; its failure may result in extended ER stress, leading to impaired neurite growth and neural differentiation (Tseng et al., 2017). Another example involves atlastin (ATL), which is responsible for ER homotypic membrane fusion and ER network formation. In PC-12 cells, ATL overexpression was reported to inhibit NGF-induced neurite outgrowth through a reduction in SOCE (Li et al., 2017). This effect may be mediated by a change in ER morphology, which is expected to affect ER-plasma membrane junctions, including a reduction in the number of STIM1 puncta, which are vital for SOCE (Li et al., 2017). STIM1 also functions in regulating growth cone growth. Pavez et al. (2019) demonstrated that STIM1 can regulate the dynamics of end-binding protein 1 and 3 (EB1/EB3), which are microtubule-binding proteins, and subsequently couple ER to microtubules in filopodia to direct growth cones through localizing Ca^2+^ signaling to filopodia. This activity can respond to guidance cues, including BDNF, netrin-1, and semaphorin-3a (Sema-3a) (Pavez et al., 2019).

### The Lysosome

The lysosome, a type of organelle responsible for the degradation of endocytosed extracellular and intracellular material through autography (Saftig and Haas, 2016), is also connected with neurogenesis. A study investigating lysosome dynamics during mouse NSC differentiation showed that the size of lysosomes decreased during this process and the lysosomes showed a tendency for transportation from the soma to newborn projections (Durso et al., 2020). In contrast, in quiescent NSCs derived from SVZ, the expression of lysosomal genes is elevated, while that of proteasomal and ribosomal genes is downregulated, suggesting that proteolysis might shift from proteasomes to lysosomes in quiescent NSCs (Kobayashi and Kageyama, 2021). Following treatment with BMP, an inducer of quiescence in proliferating NSCs, the expression of transcription factor EB (TFEB) and its dephosphorylation, which activates it, were both found to be increased, while the expression of the G1/S-specific cyclin D1 was decreased (Kobayashi et al., 2019). In contrast, exposure to bafilomycin A (BafA), an inhibitor of lysosomal function, leads to an increase in the expression of epidermal growth factor (EGF)- and Notch signaling-related genes, allowing the cell-cycle reentry of quiescent NSCs (Kobayashi et al., 2019). These results revealed that when active NSCs enter the quiescent state, the activation of lysosomal genes may play a significant role in NSC quiescence (Kobayashi et al., 2019). In later stages, the activity of the lysosome pathway is enhanced in quiescent NSCs to allow the removal of protein aggregates, thereby maintaining the ability of NSCs to reenter the activated state (Leeman et al., 2018).

The transportation of lysosomes along axons is also important for the regulation of axonal growth-cone dynamics. The knockdown of subunits of the bloc-1-related complex (BORC), which regulates the coupling of lysosomes to kinesin-1, results in reduced growth cone size and dynamics (Farias et al., 2017). Moreover, the transportation of lysosomes to axons can aid long-distance RNA transport by an RNA granule-associated phosphoinositide-binding protein called annexin A11 to guarantee protein provision to axons at sites far from the nucleus (Liao et al., 2019).

### The Endosome

Endocytosis is an essential means of regulating cell life and diseases. Additionally, the endocytosis of receptor–ligand complexes, membrane proteins, nutrients, bacteria, viruses, and other cargoes is essential for cellular defenses, cell dynamics and regulation of signaling pathways (Huotari and Helenius, 2011). Among these cargoes, receptor-ligand complexes play an important role in the regulation of neurogenesis, including neurite outgrowth, neurotransmitter release, and synaptic plasticity. Ligand/receptor complexes can be endocytosed and trafficked to lysosomes for degradation, recycled to a new location at the plasma membrane, or perform signaling functions (Yap and Winckler, 2012). For instance, mutation of the *ema* gene in Drosophila leads to defective endosomal maturation and, consequently, the failure to downregulate BMP receptor levels *via* endolysosomal degradation (Kim et al., 2010), finally resulting in abnormal synaptic overgrowth at neuromuscular junctions (Kim et al., 2010).

**FIGURE 3:**
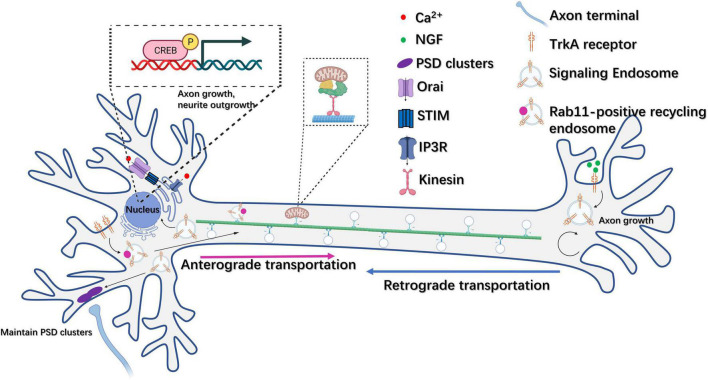
Endosome trafficking is involved in the regulation of neural plasticity. NGF-TrkA recycling endosomes can be retrogradely transported from the axon to the cell body and mediate the activation of genes related to axon outgrowth and neural differentiation, such as CREB. In addition, TrkA receptors can also be transported anterogradely from the plasma membrane at the soma to axon terminals *via* Rab11-positive recycling endosomes. Moreover, NGF-TrkA signaling endosomes can function at local axon tips to regulate axon growth. NGF-TrkA endosomes can promote synaptic maintenance through the aggregation of postsynaptic density clusters in dendrites.

The formation of recycling endosomes (REs) also plays a role in controlling neurogenesis through the delivery of surface proteins such as cell adhesion molecules (Sann et al., 2009). One such molecule, NgCAM, which resides on the axonal plasma membrane, was observed to transiently localize to the somatodendritic plasma membrane. When a dominant-negative dynamin1 (K44A) was used to downregulate endocytosis, NgCAM axon polarization was lost, indicating that REs could regulate neuronal polarity and thus direct neurogenesis (Wisco et al., 2003). The NGF family, which comprises NGF, BDNF, neurotrophin-3 (NT-3), and neurotrophin-4/5 (NT-4/5), together with their tyrosine kinase (Trk) receptors, are endocytosed as ligand-receptor complexes after binding on axon tips, and can be retrogradely transported to cell bodies or distal dendrites, or they can mediate local signaling at axons as components of signaling endosomes (Ascano et al., 2012). Specifically, NGF-TrkA signaling can initiate retrograde signaling at the nucleus, thereby promoting the activation of genes that control axon growth and neural differentiation, such as those coding for the transcription factor CREB (Riccio et al., 1997) and myocyte enhancer factor 2 (MEF2) (Figure 3; Marlin and Li, 2015). Additionally, RNA-bearing Rab7a late endosomes can interact with ribosomes, pause on mitochondria and function as the hotspots of local protein synthesis often associated with mitochondria in RGC axons (Cioni et al., 2019). It was also observed that the Charcot-Marie-Tooth disease type 2B (CMT2B)-associated mutation of Rab7a impaired local protein synthesis, mitochondria function and axon integrity (Cioni et al., 2019).

## Mitochondria and Other Organelles as Therapeutic Targets in Neurodegenerative Diseases

### Cell Organelles, Adult Neurogenesis, and Neurodegenerative Diseases

In addition to their roles in neural development, organelles such as mitochondria, ER, endosomes, and lysosomes likely also play an important role in adult neurogenesis. Indeed, there is evidence supporting that the dysfunctions of these organelles, especially mitochondria, can lead to neurodegenerative diseases.

Age-related neurodegenerative diseases such as AD, PD, Huntington’s disease (HD), and amyotrophic lateral sclerosis (ALS) represent a significant threat to human health. These disorders are often correlated with hallmarks of aging, such as DNA damage, cellular senescence, and inflammation (Hou et al., 2019). A decline in adult neurogenesis has also been strongly associated with neurodegenerative diseases, especially AD and PD. Studies involving a variety of mouse models of AD have shown that neurogenesis in the DG is reduced, NPC survival is decreased, there are fewer new β-III-tubulin-immunoreactive neurons, and neuronal differentiation of hippocampal progenitor cells is impaired in these model mice (Mu and Gage, 2011). Analysis of human AD brain samples has identified a gradual decline in the number of immature neurons in the DG concomitant with the progression of the disease (Moreno-Jimenez et al., 2019). In iPSC-derived NSCs containing a leucine-rich repeat kinase 2 (LRRK2) dominant mutation (G2019S) obtained from PD patients, deficiencies in clonal expansion and neuronal differentiation were observed after passage 16 (Liu et al., 2012). Furthermore, transgenic mice expressing the human LRRK2 G2019S mutant exhibited reduced survival and proliferation of newly generated neurons in the DG and olfactory bulb (Winner et al., 2011). Dendritic arborization and spine numbers were also decreased in newborn neurons of the DG in these mice (Winner et al., 2011). Strategies that can enhance adult neurogenesis have shown some success at relieving the symptoms of neurodegenerative diseases (Choi et al., 2018; Mishra et al., 2019; Walgrave et al., 2021).

Growing evidence has implicated organelle dysfunction in impaired adult neurogenesis. For mitochondria, the accumulation of the mutation burden in mtDNA and the dysregulation of the mitochondrial respiratory chain and mitochondrial dynamics can negatively impact adult neurogenesis. Studies have shown that adult mice with a mutation in mitochondrial replicative DNA polymerase gamma (POLG), which results in mtDNA mutagenesis, display respiratory chain deficiency and a reduction in the number of nestin-positive NSCs in the SVZ (Ahlqvist et al., 2012). Moreover, mtDNA mutation can disrupt PSC stemness and reprogramming efficiency and lead to neuron senescence through the overproduction of mitochondrial ROS (Hamalainen et al., 2015; Liang et al., 2020). Briefly, mutations in mtDNA can harm the ETC and promote ROS production, which, in turn could lead to further accumulation of mtDNA mutations (Cha et al., 2015). Furthermore, the conditional ablation of TFAM disrupts mitochondrial ETC and OXPHOS activity and impairs the neurogenesis of IPCs in 4-month-old mice (Beckervordersandforth et al., 2017). Treatment with drugs that increase the mitochondrial membrane potential also enhances the proliferation of TFAM-deficient NSPCs (Beckervordersandforth et al., 2017). Importantly, genetic inhibition of Drp1 activity can inhibit adult neurogenesis in the mouse DG under both basal and exercise conditions (Steib et al., 2014). Moreover, Drp1 overexpression enhances neuron maturation in the DG of adult mice, as evidenced by an increase in spine formation and expression of calbindin, a marker of mature DG cells (Steib et al., 2014).

As mentioned above, mitochondria dysfunction is strongly associated with neurodegeneration. A major cause of early onset familial PD is the loss of function of the mitophagy-related genes PINK1 and Parkin (Quinn et al., 2020). Mutations in PINK1 have been implicated in PD pathogenesis, which showed dysfunction of mitochondrial respiratory chain, mitochondrial depolarization and increased sensitivity to oxidative stress (Valente et al., 2004; Morais et al., 2009; Barazzuol et al., 2020). Parkin^–/–^ mice exhibit a reduction in synaptic excitability as well as deficits in behavioral paradigms sensitive to dysfunction of the nigrostriatal pathway (Goldberg et al., 2003). Disruption of the function of mitochondrial complex I led to the progressive loss of the dopaminergic phenotype in nigrostriatal axons and induced human-like parkinsonism in mice (Gonzalez-Rodriguez et al., 2021). Mitochondria dysfunction, based on the mitochondrial cascade hypothesis, is also thought to be one of the causes of AD (Swerdlow and Khan, 2009).

Significantly, metabolic regulations in mitochondria can affect pathology of AD and PD in various manners. It was found in AD patients that TCA cycle enzyme activities were downregulated, such as citrate synthase, isocitrate dehydrogenase, and α-ketoglutarate dehydrogenase complex (α-KGDHC), while the activities of succinate dehydrogenase and malate dehydrogenase were upregulated (Bubber et al., 2005; Yan et al., 2020). In particular, α-KGDHC, the rate-limiting enzyme in TCA cycle, is diminished in various neurodegenerative diseases, including AD and PD (Mizuno et al., 1994; Bubber et al., 2005). Furthermore, decrease of α-KGDHC could be observed in Aβ-expressing neurons, and prolonged reduction of α-KGDHC led to disrupted TCA cycle and OXPHOS, reduced ATP levels and overproduction of ROS (Chen et al., 2016; Teo et al., 2019). Along with dysfunction of TCA cycle, destruction of mitochondrial respiratory chain, mtDNA mutation and disruption of mitophagy can result in elevated ROS. Moreover, the intermediate amount of ROS can induce inflammatory response through activation of NF-κB and AP-1, and higher levels of ROS can lead to perturbation of the mitochondrial permeability transition pore and trigger apoptosis of neurons (Gloire et al., 2006). Under oxidative stress, NLR family pyrin domain containing 3 (NLRP3) inflammasome can be activated through Drp1 hyperactivation and glycolysis inhibition in mature oligodendrocytes, which can contribute to AD-associated neuropathology (Luo et al., 2017; Zhang et al., 2020). Additionally, increased mitochondrial ROS triggered NLRP3 inflammasome signaling in microglia and enhanced dopaminergic neurodegenerative process in PD models (Sarkar et al., 2017). Elevated ROS and inflammation in PD can further promote neurotoxicity of α-synuclein via activation of C/EBPβ/AEP signaling (Ahn et al., 2021). Significantly, mitochondrial metabolic dysfunction can trigger α-synuclein oligomerization through ROS-mediated oxidative stress and ATP depletion-driven microtubule network disruption (Esteves et al., 2009; Arduino et al., 2012). In AD cybrid cells and cortical neurons from tau mice, treatment of antioxidants targeting mitochondria can reduce accumulation of tau oligomers, suggesting that mitochondrial ROS can contribute to formation and accumulation of tau oligomers in AD (Du et al., 2022). It is also worth attention that mitochondria can affect the development of AD and PD via NAD^+^/NADH levels. NPCs differentiated from iPSCs derived from late-onset AD patient showed reduced levels of NAD^+^/NADH (Ryu et al., 2021). Due to decline of NAD^+^ levels, consequently decreased sirtuin activity can cause various disorders that lead to AD and PD pathologies. Adequate studies have revealed that Sirt1 might play the protective role against AD through suppressing inflammatory responses, facilitating mitophagy signaling pathway, reducing plaque formation and promoting clearance of accumulated tau proteins (Ye and Wu, 2021). In PD models, the α-synuclein level can be reduced through increasing NAD^+^/NADH levels and Sirt1 expression, which can subsequently enhance lysosomal function and promote elimination of α-synuclein (Dong et al., 2021). Understanding the connection among mitochondria dysfunction, adult neurogenesis impairment, and neurodegenerative diseases will likely contribute to the development of therapeutic methods for the treatment of neurodegenerative disorders.

Importantly, studies have suggested that improving mitochondrial function can ameliorate the symptoms of neurodegenerative diseases, at least to some extent, through the enhancement of adult neurogenesis. For instance, the overexpression of NeuroD1 can increase dendritic spine density and mitochondrial biogenesis in APP × PS1 adult-born neurons (Richetin et al., 2017). In primary neurons, NeuroD1 overexpression increases mitochondrial respiration concomitant with the promotion of dendritic growth and spine formation, while treatment with drugs that reduce mitochondrial membrane potential blocks NeuroD1-facilitated dendritic growth. These results suggest that restoring mitochondrial function may be critical for the promotion of neurogenesis in models of neurodegenerative disease (Richetin et al., 2017). There is also some evidence supporting that the stimulation of mitochondrial biogenesis may rescue hippocampal and cortical Aβ-induced memory impairments in AD mice through the enhancement of cell proliferation and neurogenesis (Bartolome et al., 2018). Moreover, improving the mitochondrial membrane potential and promoting mitochondrial biogenesis in the substantia nigra pars compacta (SNpc) *via* the activation of WNT/β-catenin signaling was shown to contribute to net dopaminergic neurogenesis in a PD rat model (Singh et al., 2018). Thus, pharmacological enhancement of mitochondrial function represents a promising strategy for reversing neurodegenerative diseases by facilitating adult neurogenesis.

The disruption of ER-mitochondria associations and lysosomes can also lead to neurodegenerative diseases. For instance, TAR DNA-binding protein 43 (TDP-43) can disrupt the interaction between vesicle-associated membrane protein-associated protein-B (VAPB), an ER-resident protein, and protein tyrosine phosphatase-interacting protein-51 (PTPIP51), which resides in mitochondria, thereby impairing Ca^2+^ homeostasis. The aggregation of misfolded TDP-43 proteins is a hallmark pathology of ALS and frontotemporal dementia (FTD) (Stoica et al., 2014). Moreover, loss-of-function mutations in proteins involved in autophagosome-lysosomal degradation pathways can result in neurodevelopmental disorders. Mutation in either Niemann-Pick type C 1 (NPC1) or NPC2 in late endosomes/lysosomes can result in defective cholesterol trafficking in the endocytic pathway (Nixon et al., 2008). This subsequently leads to lysosomal storage disorder and neuronal loss in the cortex, known as Niemann-Pick type C disease (Nixon et al., 2008). Additionally, a study of iPSC-derived dopaminergic neurons from PD patients revealed that the D620N mutation in vacuolar protein sorting 35 (VPS35), a member of the retromer complex, slowed the movement of Rab5a- or Rab7a-positive endosomes and led to the accumulation of α-synuclein (Bono et al., 2020).

### Cell Organelles as Therapeutic Targets in Neurodegenerative Diseases

**FIGURE 4:**
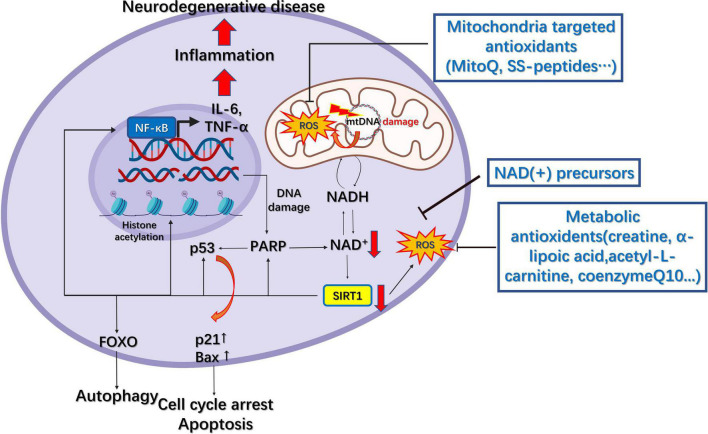
Mitochondrial pharmacology in neurodegenerative diseases. The downregulation of NAD(+) following DNA damage results in decreased SIRT1 activity, which leads to decreased deacetylation of FOXO, p53, and NF-κB and elevated levels of reactive oxygen species (ROS). This results in increased autophagy, cell cycle arrest, apoptosis, inflammation, and further DNA damage, finally leading to neurodegenerative disease. Several metabolic and mitochondria-targeted antioxidants have been developed to increase NAD(+) levels and reduce those of ROS.

That organelle dysfunction is partly responsible for various neurodegenerative diseases renders organelles potential therapeutic targets for the treatment associated conditions. Several candidates have shown the potential to target organelles, especially mitochondria, the ER, lysosomes, and endosomes (Table 1). Antioxidants, either metabolic or mitochondria-targeted, are the most commonly utilized substances for the treatment of neurodegenerative diseases given the prominent role of ROS overproduction in mitochondria-related diseases. Among the metabolic antioxidants, creatine, α-lipoic acid (LA), acetyl-L-carnitine (ALC), nicotinamide (NIC, vitamin B3), and co-enzyme Q10 (CoQ10) have shown some efficacy in treating mitochondria-related disorders (Figure 4).

**TABLE 1:** The compounds reported to be functional in models of neurodegenerative diseases targeting different organelles.

Neurodegenerative disease models	Treatment	Targeted organelles	References
Spinocerebellar ataxia type 3	Creatine	Mitochondria	Duarte-Silva et al., 2018
Glaucoma	Nicotinamide (vitamin b3)	Mitochondria (ROS)	Williams et al., 2017
Glaucoma	Nicotinamide (vitamin B3)	Mitochondria (ROS)	Tribble et al., 2021
AD	α-Lipoic acid	Mitochondria	Zhang et al., 2018
PD	α-Lipoic acid and acetyl-L-carnitine	Mitochondria	Zhang et al., 2010
AD	Omega-3 fatty acids and α-lipoic acid	Mitochondria	Shinto et al., 2014
PD	α-Lipoic acid and acetyl-L-carnitine	Mitochondria	Zaitone et al., 2012
ALS	Coenzyme Q10	Mitochondria	Matthews et al., 1998
AD	Coenzyme Q10	Mitochondria	Yang et al., 2008
AD	(3Carboxypropyl)triphenyl-phosphonium bromide-conjugated 1,2-distearoyl-sn-glycero-3-phosphoethanolamine-N-[amino(polyethylene glycol)-2000]-functionalized molybdenum disulfide quantum dots (TPP-MoS2 QDs)	Mitochondria	Ren et al., 2020
HD	MitoQ and SS31	Mitochondria	Yin et al., 2016
AD	MitoQ, SS31 and anti-aging agent resveratrol	Mitochondria	Manczak et al., 2010
PD	MitoQ	Mitochondria	Xi et al., 2018
AD	MitoQ	Mitochondria	Young and Franklin, 2019
ALS	MitoQ	Mitochondria	Miquel et al., 2014
PD	Kinetin	Mitochondria	Hertz et al., 2013
HD	Mdivi-1	Mitochondria	Manczak and Reddy, 2015
AD	Mdivi-1 and SS31	Mitochondria	Reddy et al., 2018
AD	Dantrolene (RyR inhibitor)	ER	Chakroborty et al., 2012
AD	Dantrolene (RyR inhibitor)	ER	Oules et al., 2012
PD	SKF-96365	ER	Chen et al., 2013
HD	EVP4593	ER	Wu et al., 2016
HD	EVP4593	ER	Vigont et al., 2015
HD	Tetrahydrocarbazoles (6-bromo-N-(2-phenylethyl)-2,3,4,9-tetrahydro-1H-carbazol-1-amine hydrochloride)	ER	Czeredys et al., 2017
AD	Lonafarnib (farnesyltransferase inhibitor)	Lysosome	Hernandez et al., 2019
AD	VER-1550089 (heat shock cognate 70 inhibitor)	Lysosome	Yang and Tohda, 2018
Niemann pick type C disease	CGS21680 (A_2A_R agonist)	Lysosome	De Nuccio et al., 2019

The creatine/phosphocreatine system serves as a regulator of cellular energy balance through creatine kinases (CKs) coupled to ATP production, while cytosolic creatine can also maintain mitochondrial CKs in an octameric conformation, which inhibits the opening of mitochondrial permeability transition pore (Adhihetty and Beal, 2008). Furthermore, creatine supplementation can rescue the expression of the mitochondrial mass marker Porin as well as that of antioxidant enzymes in a mouse model of spinocerebellar ataxia type 3, a hereditary neurodegenerative disease (Duarte-Silva et al., 2018). LA is an antioxidant that can also function as the co-enzyme for mitochondrial pyruvate dehydrogenase and α-ketoglutarate dehydrogenase, and its supplementation can effectively inhibit tau hyperphosphorylation, caspase-dependent apoptosis, peroxidation, and inflammation in tau P301S transgenic mouse models of AD (Zhang et al., 2018). Additionally, the application of LA and ALC can effectively attenuate mitochondrial dysfunction in a human neuroblastoma SK-N-MC cell model of rotenone-induced PD (Zhang et al., 2010). Another commonly used antioxidant, CoQ10, the co-factor of the ETC, can accept electrons from complex I and II, thereby inhibiting lipid peroxidation and protecting mitochondrial proteins and DNA from oxidative damage (Arenas-Jal et al., 2020). A study has shown that oral administration of CoQ10 markedly reduced striatal lesions and increased mitochondrial concentrations in the brains of 3-nitropropionic acid ALS model rats (Matthews et al., 1998). CoQ10 treatment can also rescue Aβ_1–40_-induced mitochondrial alterations in the brain and attenuate the reduction in oxidative phosphorylation efficiency (Moreira et al., 2005). During the aging process, nicotinamide adenine dinucleotide [NAD(+)] levels decline owing to an increase in DNA damage and inflammation, consequently leading to decreased sirtuin activity in the nucleus and mitochondria (Imai and Guarente, 2014; Verdin, 2015). Reduced Sirt1 activity can further lead to increased NF-κB activation and decreased FOXO3a activity, which causes neural inflammation and cognitive dysfunction (Verdin, 2015). In mouse models of glaucoma, oral administration of the NAD(+) precursor nicotinamide significantly reduces vulnerability to glaucoma, a neurodegenerative disease that can result in vision loss, by supporting mitochondrial health and metabolism (Williams et al., 2017).

Although the antioxidants mentioned above cannot precisely increase the antioxidant levels within mitochondria, mitochondria-targeted antioxidants have been developed to meet this requirement. The commonly applied strategy for targeting mitochondria is to make compounds selectively accumulate within these organelles, for example, through conjugating molecules to lipophilic cations, such as triphenylphosphonium (TPP), or using mitochondria-targeted peptides, including mitochondria-penetrating peptides (MPPs) and Szeto–Schiller (SS) peptides (Smith et al., 2012), such as the conjugation of TPP to antioxidants such as co-enzyme Q or the α-tocopherols MitoQ and MitoVitE. SS peptides are cell-permeable tetrapeptides that include four members, namely, SS31, SS02, SS19, and SS20. SS31 has a sequence motif that allows it to target mitochondria, scavenge free radicals such as H_2_O_2_ and ONOO^–^, and inhibit lipid peroxidation (Yin et al., 2016). Treating striatal neurons carrying stably expressed mutant huntingtin (Htt) (STHDhQ111/Q111) with SS31 and MitoQ upregulated synaptophysin and PSD95 expression in neurons and largely normalized mitochondrial function (Yin et al., 2016). Additionally, MitoQ was reported to prolong life span, delay Aβ-induced paralysis, and protect complexes I and IV in *Caenorhabditis elegans* expressing human Aβ, although it failed to reduce mtDNA oxidative damage (Ng et al., 2014).

Components that can modulate mitochondrial dynamics are also considered potential candidates for the treatment of neurodegenerative diseases. Hertz et al. (2013) found that the ATP analog kinetin triphosphate (KTP) can increase the activity of PINK1 harboring the PD-related variant G309D, resulting in enhanced recruitment of Parkin to depolarized mitochondria. Kinetin riboside ProTides can also promote PINK1 activity in cells (Osgerby et al., 2017). Importantly, Mdivi-1, a mitochondria division and Drp1 inhibitor, can reversibly inhibit complex I and attenuate pathological ROS production (Bordt et al., 2017). One study showed that Mdivi-1 exerts a protective effect on mitochondria and synapses in striatal neurons stably expressing mutant Htt (STHDhQ111/Q111) (Manczak and Reddy, 2015). Mdivi-1 and SS31 treatment also exert synergistic protective effects against mouse neuroblastoma (N2a) cells transfected with mutant AβPP cDNA (Swedish and Indiana mutations), resulting in lower Aβ40 and Aβ42 levels, reduced mitochondria dysfunction, and increased mtDNA copy number and cell survival (Reddy et al., 2018).

Besides mitochondria, strategies targeting the ER also show promise in the treatment of AD, PD, and HD. SOCE dysregulation can perturb intracellular Ca^2+^ signaling in neurons, leading to ER stress, synaptic loss, and cognitive decline (Secondo et al., 2018). The application of dantrolene, an inhibitor of the ER-localized ryanodine receptors (RyR), normalized ER Ca^2+^ signaling within somatic and dendritic compartments in hippocampal slices of early and late-stage AD mice (Chakroborty et al., 2012). Regarding PD treatment, the *in vitro* application of SKF-96365, a non-specific inhibitor of SOCE, reduced intracellular Ca^2+^ overload and inhibited homer1-mediated ER Ca^2+^ release in a PC12 cell model of neurotoxin N-methyl-4-phenylpyridinium [MPP(+)]-induced PD (Chen et al., 2013). In addition, other inhibitors of the SOCE machinery, such as EVP4593, have also exhibited therapeutic potential against HD *via* the restoration of cellular Ca^2+^ homeostasis (Vigont et al., 2015; Wu et al., 2016). The development of compounds targeting lysosomes has also provided some benefit in the treatment of neurodegenerative diseases (Bonam et al., 2019). Lonafarnib, a farnesyltransferase inhibitor, was reported to ameliorate tau pathology and attenuate behavioral abnormalities in a rTg4510 mouse model through the enhancement of lysosome-mediated protein degradation (Hernandez et al., 2019). Finally, the administration of the adenosine A_2A_ receptor (A_2A_R) agonist CGS21680 rescued the abnormal levels of autophagy and cholesterol accumulation in late endosomes/lysosomes in a Niemann Pick type C (NPC) oligodendrocyte progenitor model (De Nuccio et al., 2019). Although only relatively few lysosome-targeted drugs have been identified to date, targeting lysosomes remains a promising strategy for use in the treatment of neurodegenerative diseases.

## Discussion

Future studies should focus on determining whether metabolic reprogramming targeting mitochondria can help to generate functional neurons. For instance, suppressing PI3K using LY294002 can increase the mitochondrial membrane potential and promote the differentiation of mNSCs and human dermal stem cells (hDSCs) into TUJ1-positive, neuron-like cells with electrophysiological activity (Liu et al., 2019). During this process, metabolism switches from glycolysis to OXPHOS with a parallel increase in the mitochondrial membrane potential and mitochondrial ROS production (Liu et al., 2019). Moreover, mitochondria-associated proteins may serve as markers for assessing the extent of neuroectodermal differentiation. The mRNA expression of CHCHD2, a mitochondrial protein, was found to be decreased in human iPSCs relative to that in human ESCs (Zhu et al., 2016). This difference will reduce the ability of human iPSCs to differentiate along a neural lineage as lower CHCHD2 levels equate to reduced sequestration of SMAD4 in mitochondria and, consequently, a failure to inhibit the transforming growth factor-β (TGF-β) signaling pathway (Zhu et al., 2016). This implies that mitochondrial proteins may also represent promising targets for enhancing neural differentiation in human iPSCs.

Although several drugs that target organelles have been developed, it is necessary to identify additional drugs with improved safety and efficiency. With the rapid development of high-throughput screening technology, the design of probes precisely targeting specific organelles can greatly improve drug screening efficiency. For example, Cen et al. (2020) screened 2,024 drug candidates or FDA-approved drugs and identified UMI-77 as an activator of mitophagy with potential for use in AD treatment. During the screening process, the authors used a HEK293T cell line stably expressing mt-Keima (Cen et al., 2020). Keima is a coral-derived protein that exhibits shorter-wavelength (green) excitation in mitochondria (pH 8.0) and longer-wavelength (red) excitation in lysosomes (pH 4.5), and is resistant to lysosome degradation (Sun et al., 2017). Recently, another mitophagy probe was developed, called mito-SARI, which displayed improved resistance to fixation. This probe was used to identify T-271, an enhancer of mitophagy, in a large-scale-based high-throughput screen of 76,000 candidates (Katayama et al., 2020). This compound could also induce mitophagy in a Parkin-dependent manner without damaging mitochondria, rendering it and its derivatives potential therapeutic options for the treatment of neurodegenerative diseases (Katayama et al., 2020).

In conclusion, it is foreseeable that targeting organelles for neural conversion in regenerative medicine and drug screening will lead to improvements in the treatment of neurodegenerative disorders.

## Author Contributions

SZ and HL conceived and designed the review. SZ drafted. HQ, HL, JZ, and SZ revised. HQ, JZ, ZQ, HL, and SZ discussed. All authors contributed to the article and approved the submitted version.

## Conflict of Interest

The authors declare that the research was conducted in the absence of any commercial or financial relationships that could be construed as a potential conflict of interest.

## Publisher’s Note

All claims expressed in this article are solely those of the authors and do not necessarily represent those of their affiliated organizations, or those of the publisher, the editors and the reviewers. Any product that may be evaluated in this article, or claim that may be made by its manufacturer, is not guaranteed or endorsed by the publisher.
